# Supplementary material for: The TRIM37 variant rs57141087 contributes to triple-negative breast cancer outcomes in Black women
Source: EMBO Rep. 2024 Nov 29;26(1):245–72. doi: 10.1038/s44319-024-00331-2 (PMC11723928; doi:10.1038/s44319-024-00331-2)
Supplement: Supplementary file 1 — Table EV1 [file 44319_2024_331_MOESM1_ESM.docx]

Table EV1: Characteristics of tumor samples and normal breast tissue utilized in the study.

| Parameter | Number of samples | Black women | White women |
| --- | --- | --- | --- |
| ***TRIM37 expression in TNBC samples (n=319)*** | | | |
| Tumor Stage I | 71 | 28 (39.44%) | 43 (60.56%) |
| Tumor Stage II-IV | 248 | 114 (45.97%) | 134 (54.03%) |
| TRIM37-Low | 125 | 63 (50.4%) | 62 (49.6%) |
| TRIM37-High | 194 | 79 (40.72%) | 115 (59.28%) |
| ***TRIM37 copy number in TNBC samples (n=169)*** | | | |
| Deletion | 90 | 33 (52.38%) | 57 (53.77%) |
| Amplification | 75 | 29 (46.03%) | 46 (43.40%) |
| No change | 4 | 1 (1.59%) | 3 (2.83%) |
| ***Survival analyses for TNBC patients (n=216)*** | | | |
| TRIM37-Low | 79 | 37 (46.84%) | 42 (53.16%) |
| TRIM37-High | 137 | 49 (35.77%) | 88 (64.23%) |
| ***Normal breast samples (n=549)*** | | | |
| Age (years)  ≤35  >35 | 115  434 | 27 (23.48%)  92 (21.20%) | 88 (76.52%)  342 (78.80%) |
| TRIM37 expression  Low-  High- | 279  270 | 48 (17.20%)  71 (26.30%) | 231 (82.80%)  199 (73.70%) |
| ***Cancer-free breast tissue from women at risk (n=178)*** | | | |
| Age (years)  ≤35  >35 | 69  109 | 12 (17.40%)  18 (16.51%) | 57 (82.60%)  91 (83.49%) |
| Cancer risk  Average  High | 106  72 | 21 (19.81%)  9 (12.5%) | 85 (80.19%)  63 (87.5%) |
| BMI  Healthy  Overweight  Obese | 68  72  38 | 8 (11.76%)  16 (22.22%)  6 (15.79%) | 60 (88.24%)  56 (77.78%)  32 (84.21%) |
| Menopausal status  Pre-  Post- | 114  64 | 20 (17.54%)  10 (15.63%) | 94 (82.46%)  54 (84.37%) |
| TRIM37 expression  Low-  High- | 95  83 | 12 (12.63%)  18 (21.69%) | 83 (87.37)  65 (78.31) |
| Parity  No  Yes | 43  135 | 4 (9.30%)  26 (19.26%) | 39 (90.70%)  109 (80.74%) |
