# Supplementary material for: The TRIM37 variant rs57141087 contributes to triple-negative breast cancer outcomes in Black women
Source: EMBO Rep. 2024 Nov 29;26(1):245–72. doi: 10.1038/s44319-024-00331-2 (PMC11723928; doi:10.1038/s44319-024-00331-2)
Supplement: Supplementary file 2 — Table EV2 [file 44319_2024_331_MOESM2_ESM.docx]

Table EV2: Characteristics of paraffin-archived normal breast tissue.

| ***Cancer-free paraffin-archived normal breast tissue (n=6)*** | | | | | | |
| --- | --- | --- | --- | --- | --- | --- |
| Parameter | Sample #1 | Sample #2 | Sample #3 | Sample #4 | Sample #5 | Sample  #6 |
| Racial identity | Black | Black | Black | White | White | White |
| Age (<45 Years) | Yes | Yes | Yes | Yes | Yes | Yes |
| Menopausal status | Pre- | Pre- | Pre- | Pre- | Pre- | Pre- |
| Parity | Yes | Yes | Yes | Yes | Yes | Yes |
| Diagnosed with BC | No | No | No | No | No | No |
| Ever smoked | Yes | No | No | Yes | No | No |
| Test Positive for Genetic Risk | No | No | No | N/A | No | No |
| BRCA1 | No | No | No | No | No | No |
| BRCA2 | No | No | No | No | No | No |
| Blood Relatives Cancer | N/A | No | No | Yes | No | Yes |
| Height (ft) | 5 | 5 | 5 | 5 | 5 | 5 |
