# Supplementary figures and images for: The TRIM37 variant rs57141087 contributes to triple-negative breast cancer outcomes in Black women
Source: EMBO Rep. 2024 Nov 29;26(1):245–72. doi: 10.1038/s44319-024-00331-2 (PMC11723928; doi:10.1038/s44319-024-00331-2)

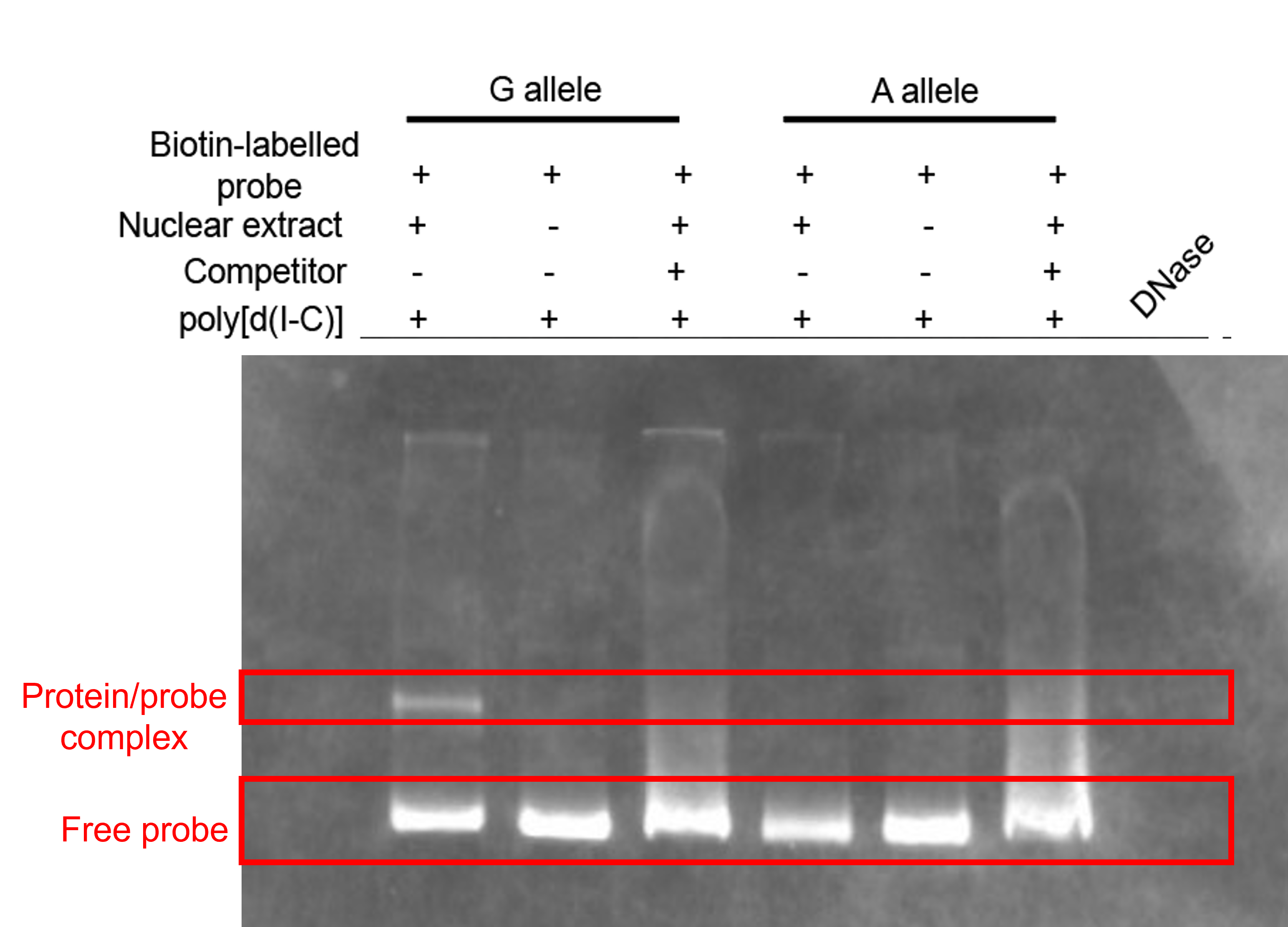

Supplement: Supplementary file 7 — Source data Fig. 4 [file 44319_2024_331_MOESM7_ESM.zip › Figure_4/Figure_4C/Figure_4C.tif]

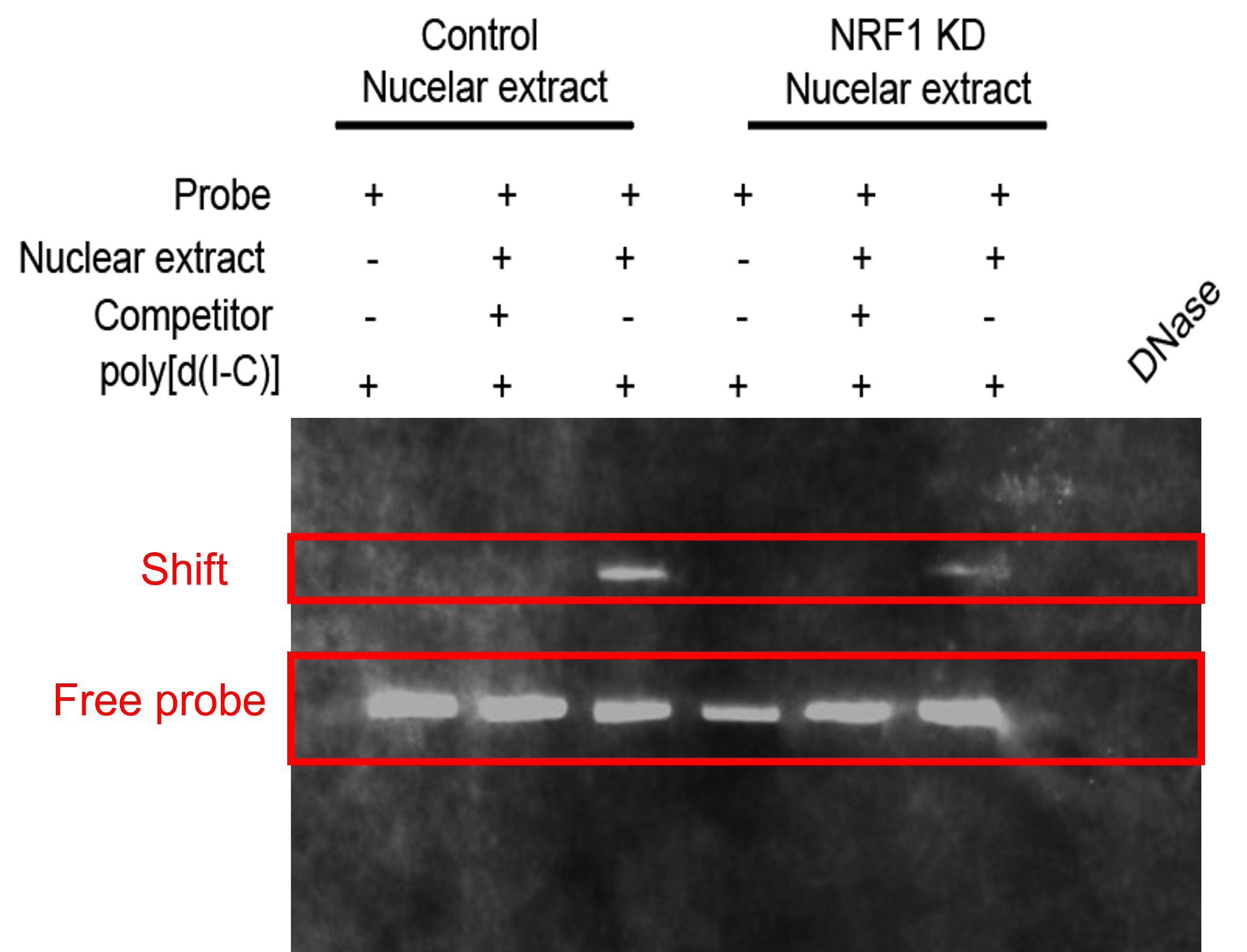

Supplement: Supplementary file 7 — Source data Fig. 4 [file 44319_2024_331_MOESM7_ESM.zip › Figure_4/Figure_4G/Figure_4G.tif]

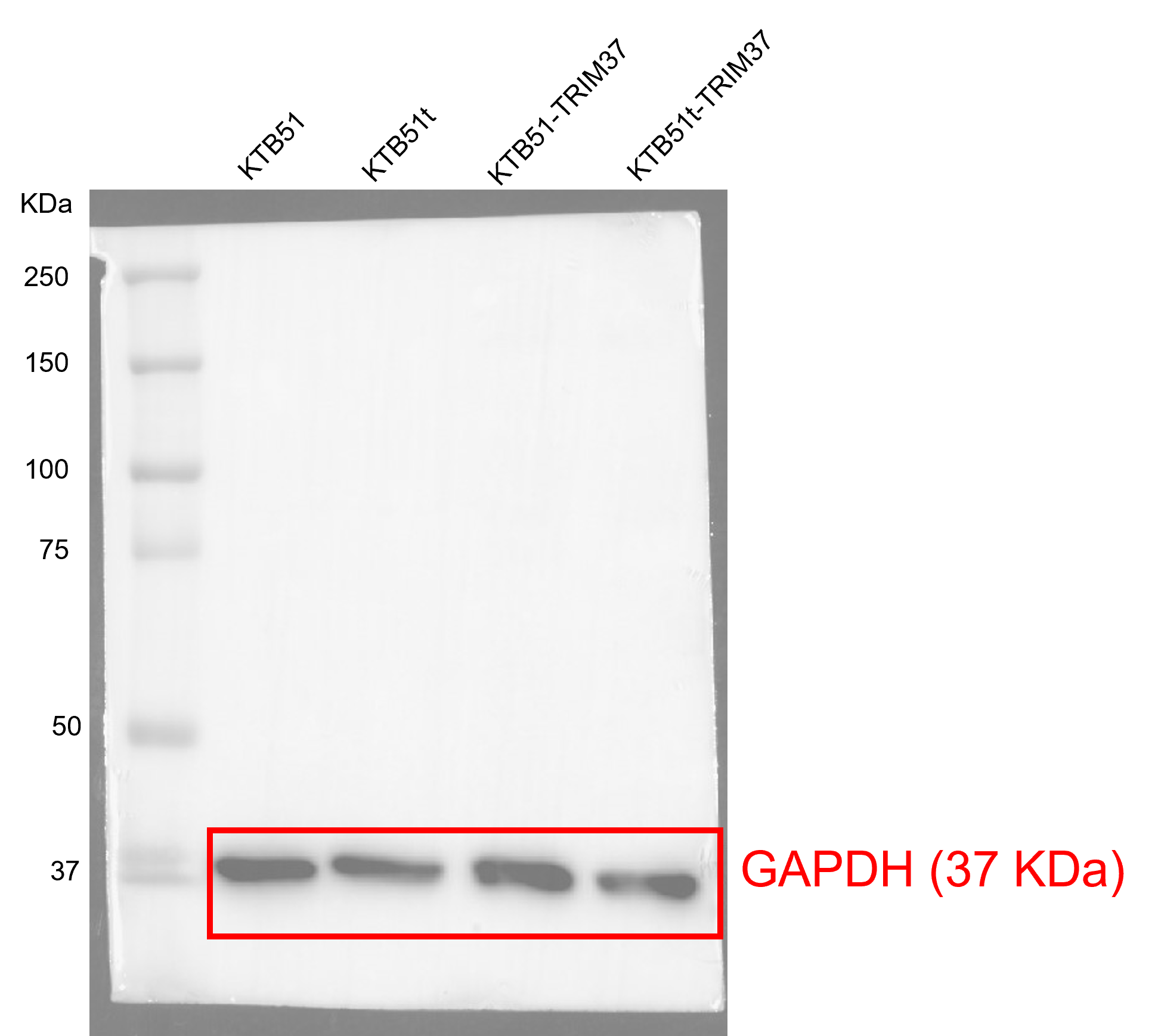

Supplement: Supplementary file 8 — Source data Fig. 5 [file 44319_2024_331_MOESM8_ESM.zip › Figure_5/Figure_5A/Figure_5A_GAPDH.tif]

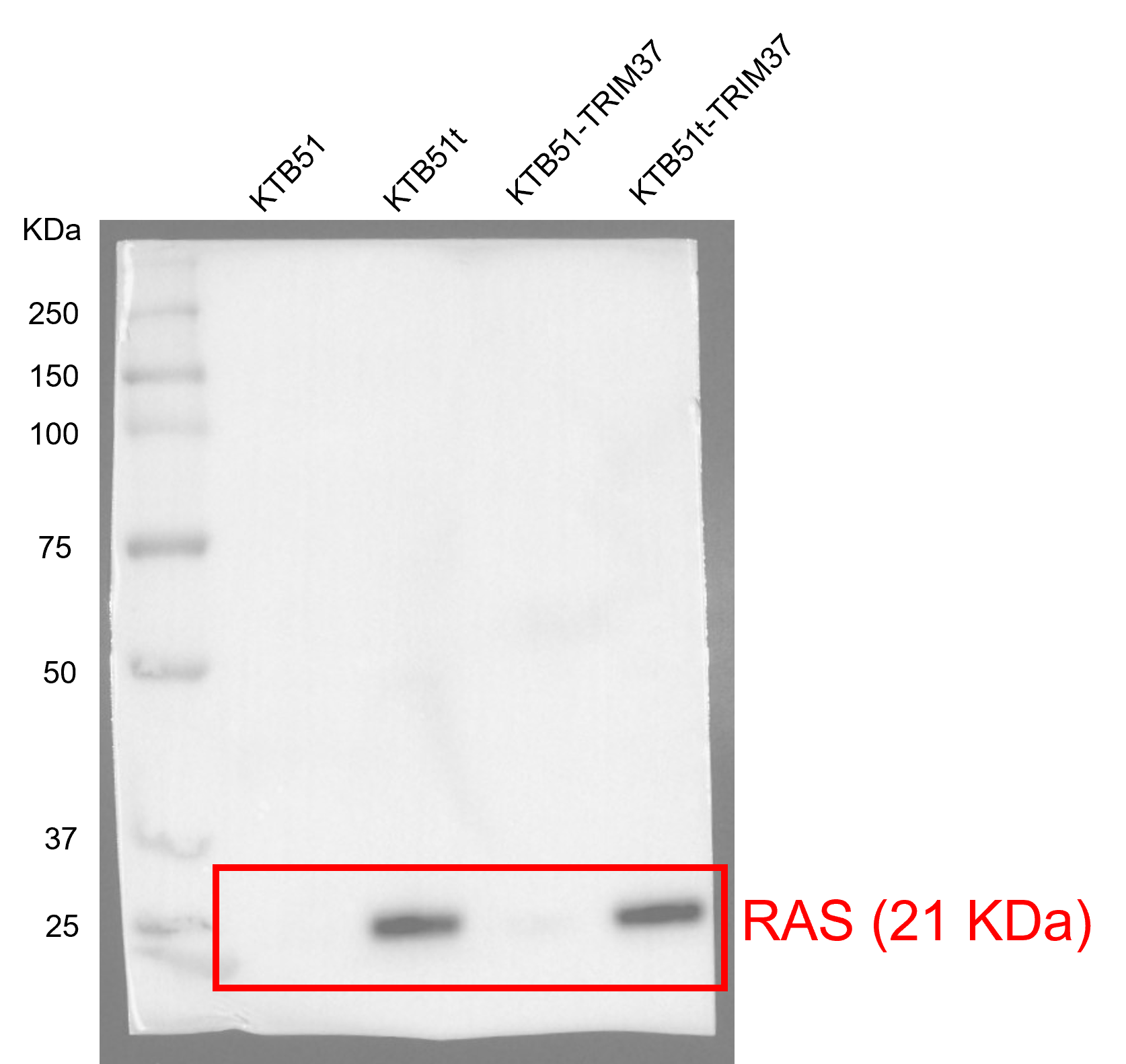

Supplement: Supplementary file 8 — Source data Fig. 5 [file 44319_2024_331_MOESM8_ESM.zip › Figure_5/Figure_5A/Figure_5A_RAS.png]

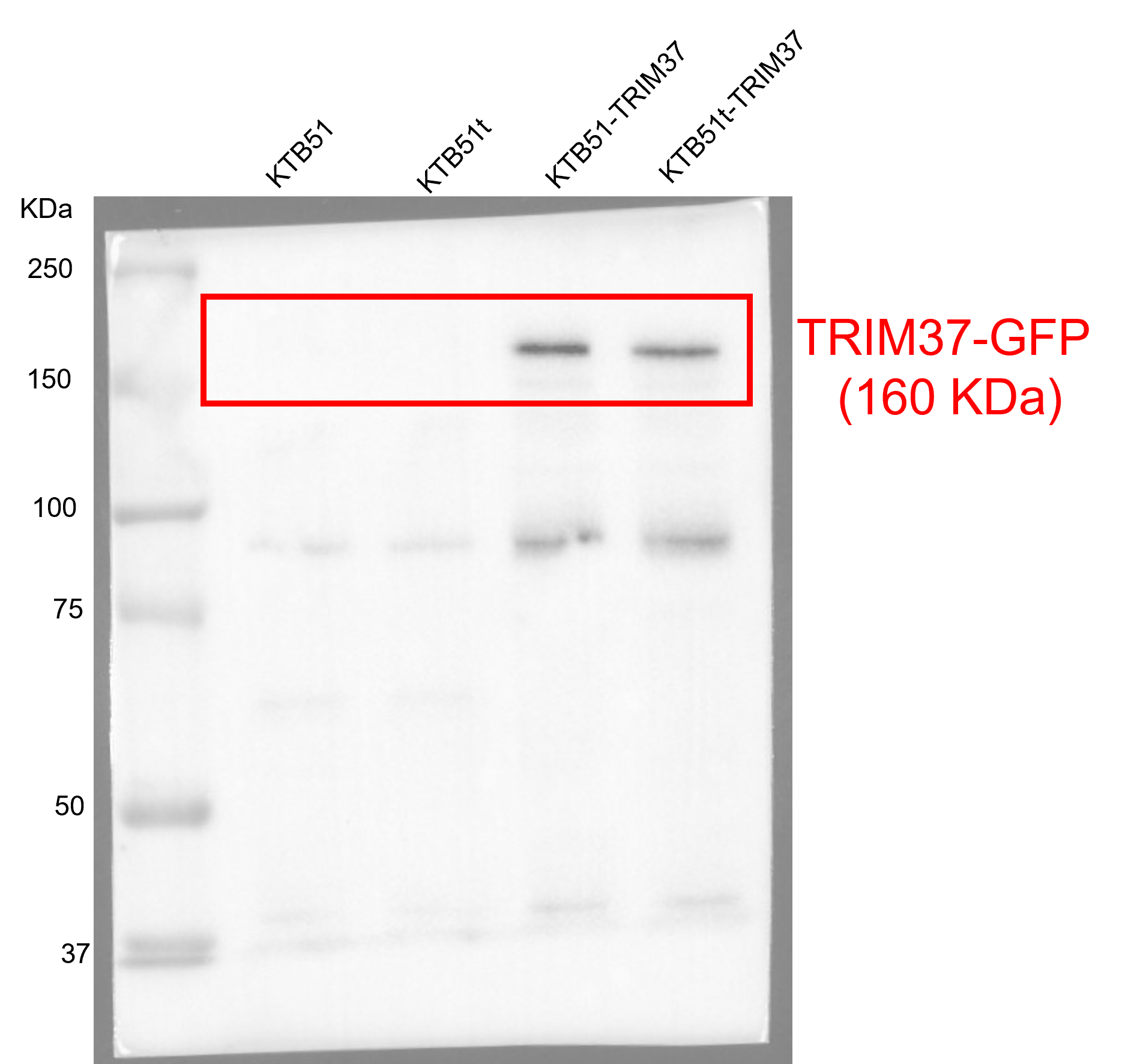

Supplement: Supplementary file 8 — Source data Fig. 5 [file 44319_2024_331_MOESM8_ESM.zip › Figure_5/Figure_5A/Figure_5A_TRIM37.tif]

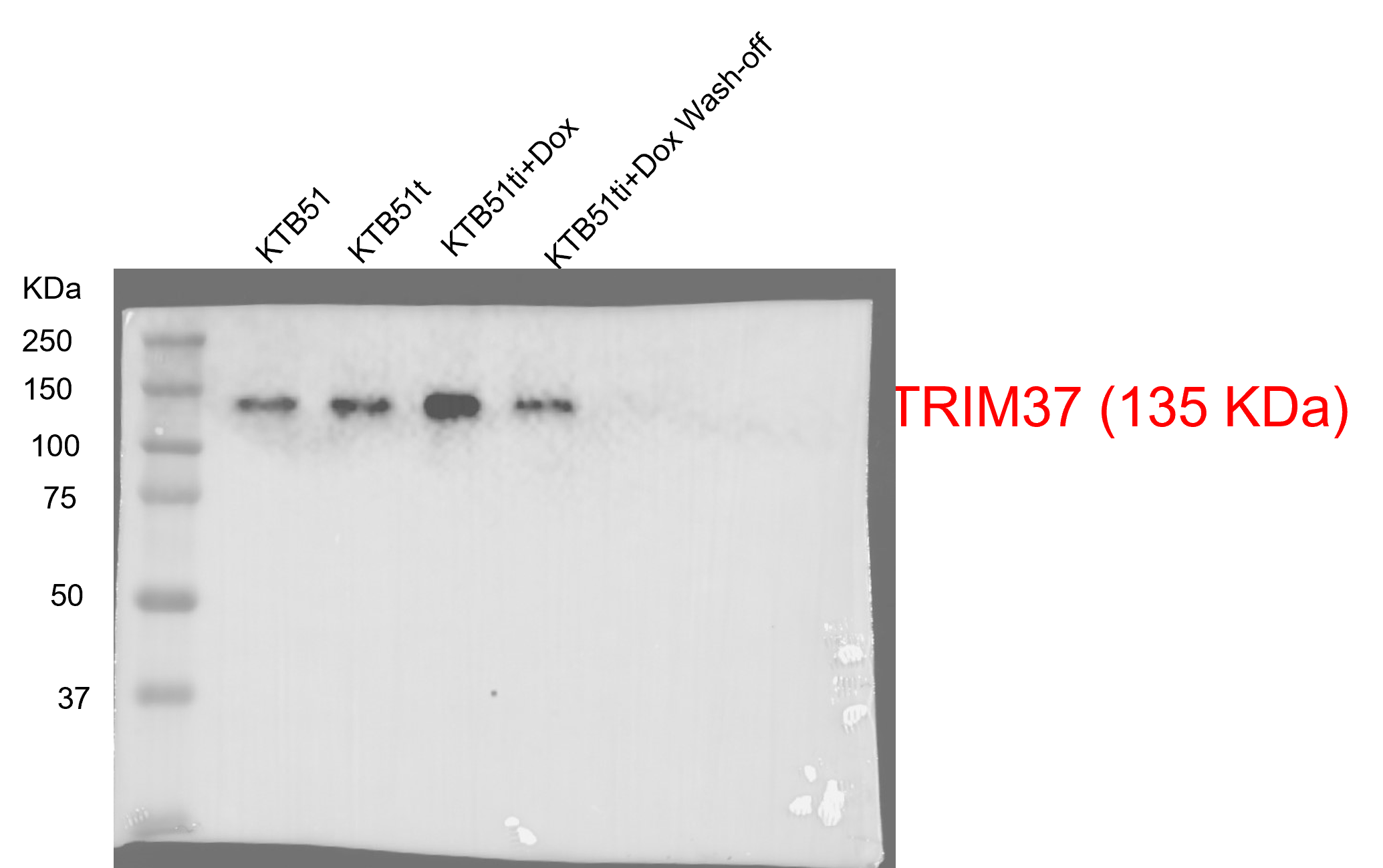

Supplement: Supplementary file 9 — Source data Fig. 6 [file 44319_2024_331_MOESM9_ESM.zip › Figure_6/Figure_6A/Figure_6A_TRIM37.tif]

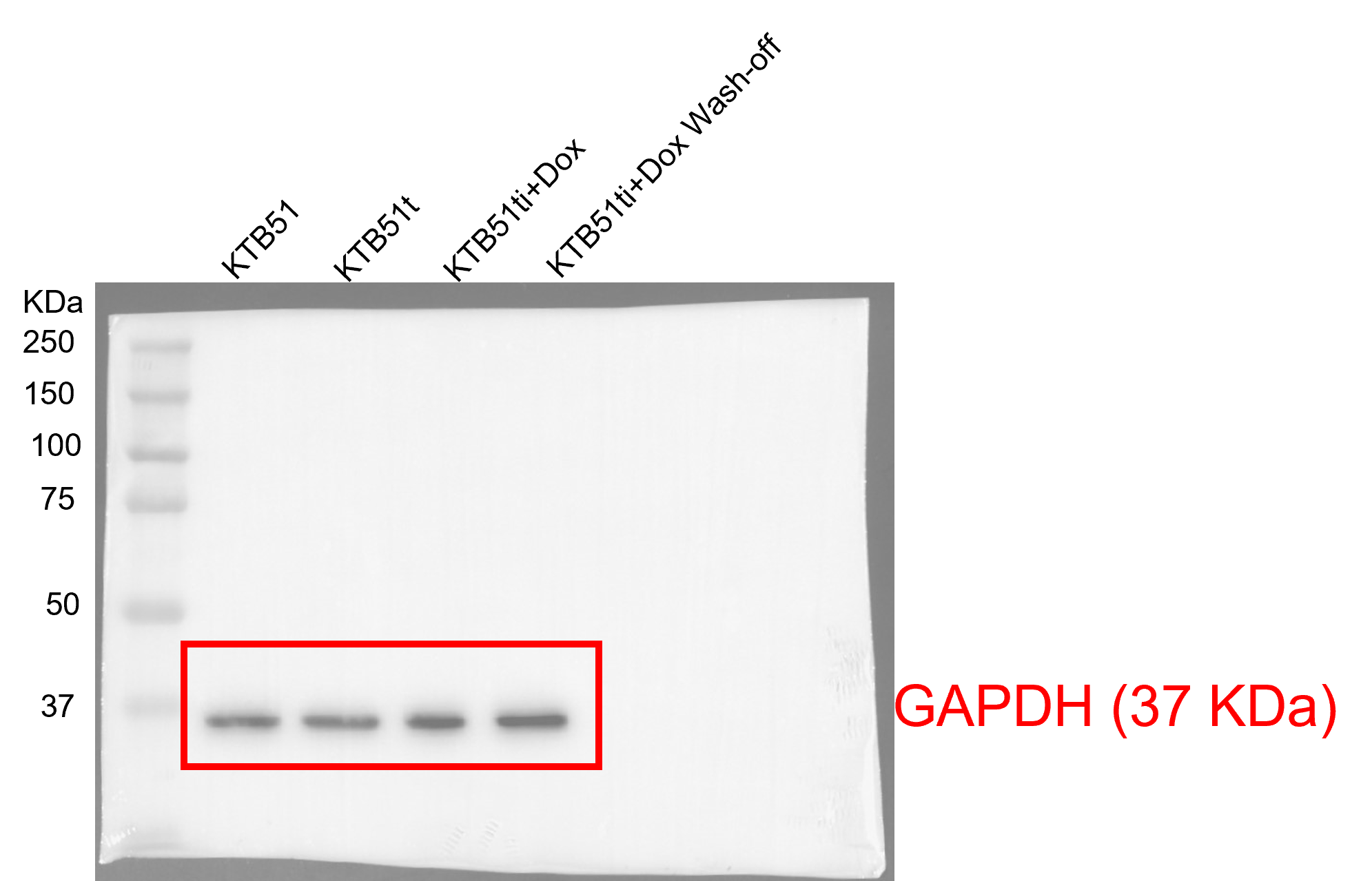

Supplement: Supplementary file 9 — Source data Fig. 6 [file 44319_2024_331_MOESM9_ESM.zip › Figure_6/Figure_6A/Figure_6A_GAPDH.tif]
