# Supplementary material for: The TRIM37 variant rs57141087 contributes to triple-negative breast cancer outcomes in Black women
Source: EMBO Rep. 2024 Nov 29;26(1):245–72. doi: 10.1038/s44319-024-00331-2 (PMC11723928; doi:10.1038/s44319-024-00331-2)
Supplement: Supplementary file 10 — Expanded View Figures [file 44319_2024_331_MOESM10_ESM.pdf]

Expanded View Figures

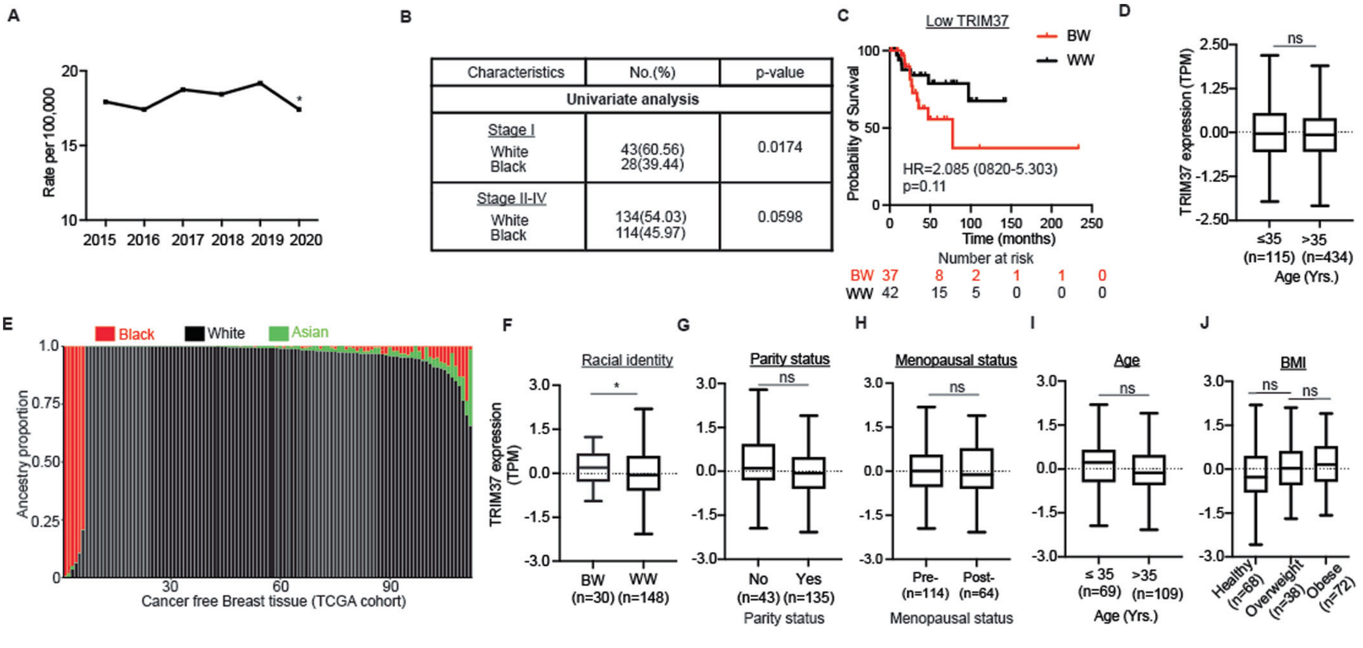

Figure EV1. Ancestry-specific differences in TNBC survival and incidence.

(**A**) Breast cancer incidence rate per 100,000 in women, excluding Hispanics, from 2015-2020 Surveillance, Epidemiology, and End Results (SEER) data. $^{*}P = 0.038$, proportional test. The number of samples is indicated. (**B**) Univariate analyses of relationships between *TRIM37* and racial identity in Stage I ($n = 71$) and Stage II–IV ($n = 248$) TNBC patients (GSE142731, Data ref: Saleh et al, 2021; PRJNA704957, Data ref: NCBI Sequence Read Archive PRJNA704957, 2021, and TCGA, Data ref: The Cancer Genome Atlas Program (TCGA-Breast)) (**C**) Kaplan–Meier survival curve showing overall survival for the BW and WW TNBC patients with low *TRIM37* expression (GSE39004, Data ref: Tang et al, 2018; TCGA, Data ref: The Cancer Genome Atlas Program (TCGA-Breast), and GSE18229, Data ref: Prat et al, 2010). The number of surviving patients at 0, 50, 100, 150, 200, and 250-month time points is indicated below the graph. $^{ns}P = 0.11$, log-rank test. The number of surviving patients at 0, 50, 100, 150, 200, and 250-month time points are indicated. (**D**) Box plot for *TRIM37* expression in the normal breast tissue by age, below or equal to 35 ($n = 115$) vs. greater than 35 years ($n = 434$) (GSE164641, Data ref: Marino et al, 2022"; GSE111601, Data ref: Sun et al, 2018; GTEX, Data ref: The Genotype-Tissue Expression (GTEx), and TCGA, Data ref: The Cancer Genome Atlas Program (TCGA-Breast)). $^{ns}P = 0.95$, t test. The boxed areas span the first to the third quartile, with the central line representing the median expression changes for each group. Outliers from the boxplots are not displayed. The whiskers represent the 15th and 85th percentiles. (**E**) Estimated genetic ancestry distribution for self-reported BW ($n = 6$) and WW ($n = 103$) in the TCGA cohort (Data ref: The Cancer Genome Atlas Program (TCGA-Breast)). Each column represents an individual in the cohort, and the estimated proportion of African, European, and Asian ancestry is shown on the y-axis. (**F–J**) Box plot for *TRIM37* expression in the breast tissue from women stratified by racial identity (Black vs. White), parity (No vs. yes), menopausal status (Pre-vs. Post), age (below or equal to 35 vs. greater than 35 years) and BMI (Healthy vs. overweight vs. obese) (GSE164641, Data ref: Marino et al, 2022). For race, $^{*}P = 0.05$, parity, $^{ns}P = 0.087$, menopausal status, $^{ns}P = 0.52$, age, $^{ns}P = 0.3$, and BMI, $^{ns}P = 0.17$, *t* test. The number of samples is indicated. The boxed areas span the first to the third quartile, with the central line representing the median expression changes for each group. Outliers from the boxplots are not displayed. The whiskers represent the 15th and 85th percentiles.

© The Author(s)

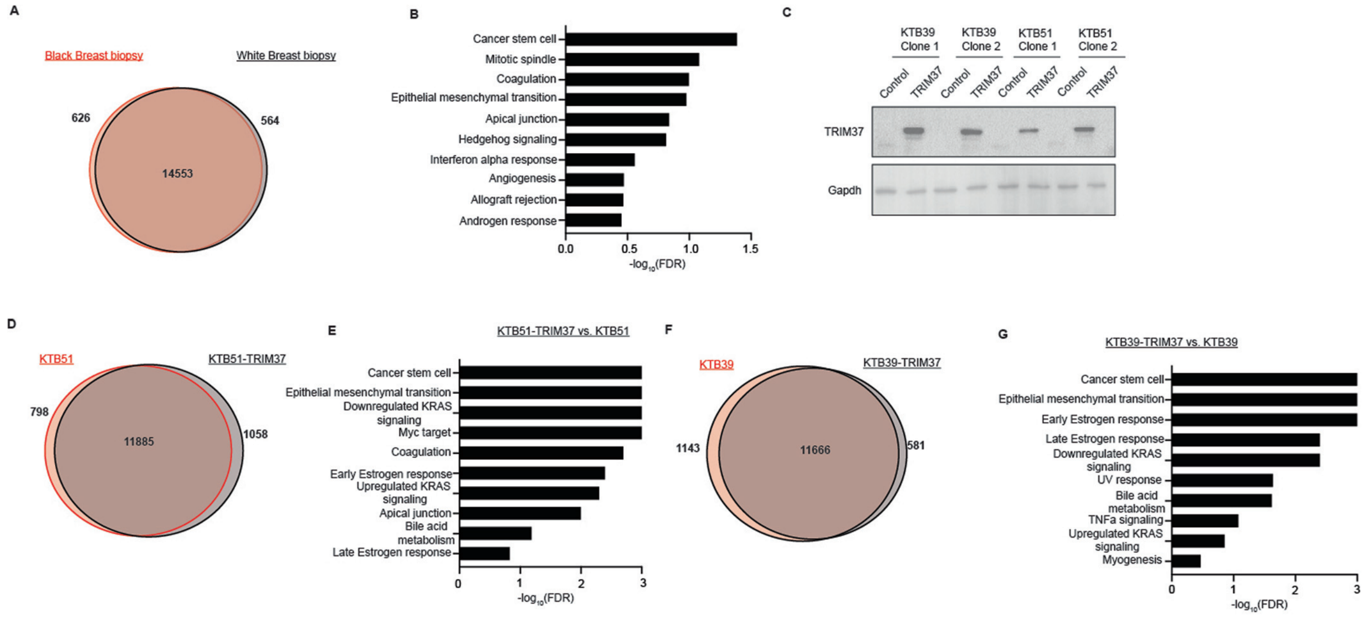

Figure EV2. ***TRIM37* drives cancer stemness and epithelial to mesenchymal transition phenotype in Black breast epithelial cells.**

(**A**) Venn diagram showing the overlap between DEGs in the cancer-free breast tissue samples of parous and premenopausal BW ($n = 3$; ages <45) and WW ($n = 3$; ages <45). (**B**) The top ten pathways enriched in the cancer-free breast tissue samples of BW relative to WW identified by GSEA are shown. (**C**) Immunoblot analysis of control and *TRIM37* overexpressing KTB39 and KTB51 cells. Two clones for each cell line are shown. Gapdh was used as a loading control. (**D**) Venn diagram showing the overlap between differentially expressed genes in control and *TRIM37* overexpressing KTB51 cells. (**E**) The top ten pathways significantly enriched in *TRIM37* overexpressing KTB51 cells relative to control cells identified by GSEA are shown. (**F**) Venn diagram showing the overlap between differentially expressed genes in control and *TRIM37* overexpressing KTB39 cells. (**G**) The top ten pathways enriched in *TRIM37* overexpressing KTB39 cells relative to control cells identified by GSEA are shown.

 © The Author(s)

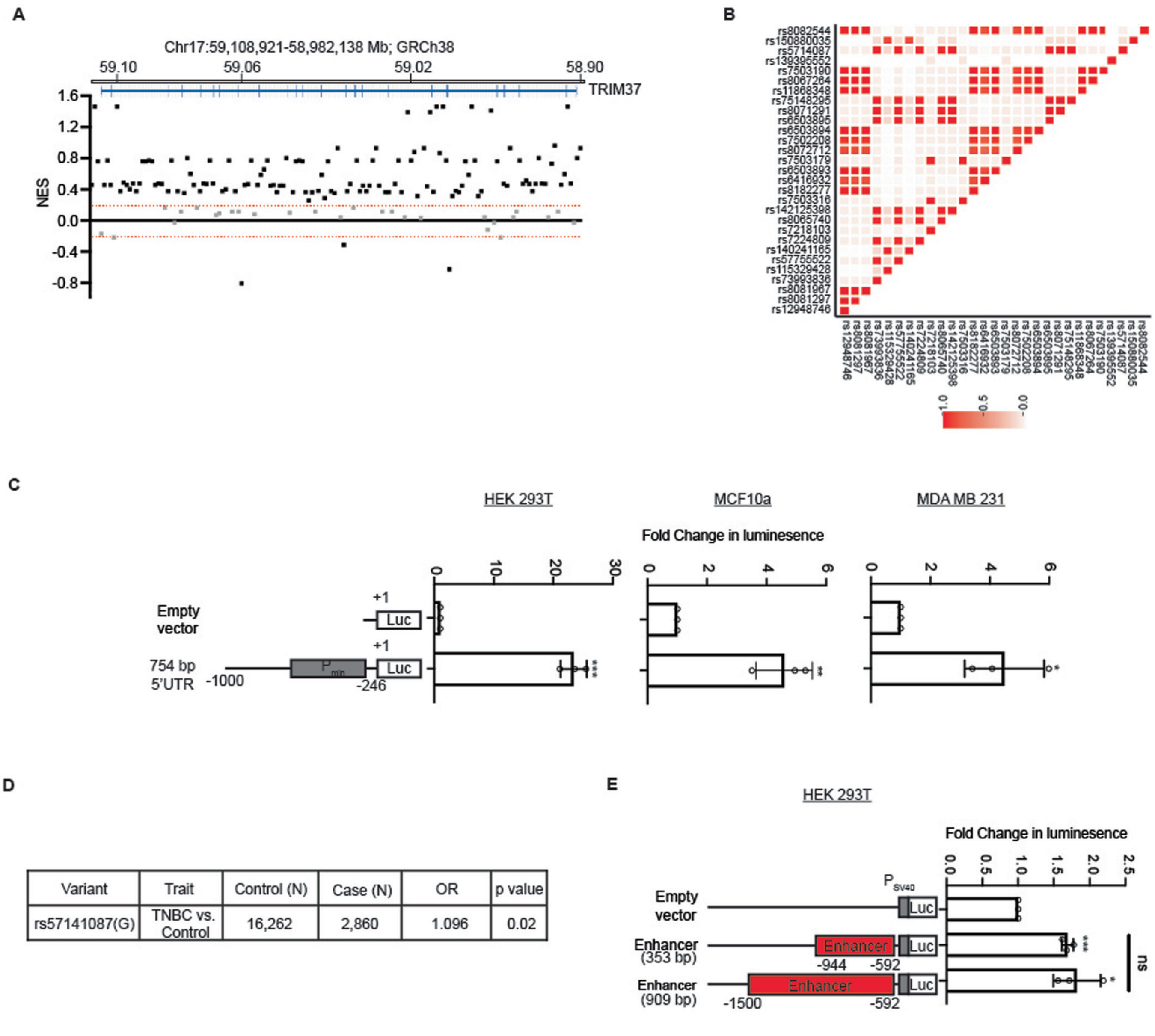

Figure EV3. rs57141087 increases *TRIM37* expression through modulation of enhancer function.

(**A**) Distribution of SNPs across the TRIM37 gene (Chr17:59,108,921–58,982,138 Mb) and their association with *TRIM37* expression in the breast tissue from GTEx. (**B**) Linkage disequilibrium (r^2) heatmap of SNPs in TRIM37 gene in African (Yoruba, Luhya, Gambia, Mende, Esan, Americans of African Ancestry, African Caribbean) population from the 1000 Genome project data. (**C**) Luciferase reporter assays measure the promoter performance of the TRIM37 minimal promoter (Chr17: 59,107,052–59,106,707) in HEK293T, MCF10a, and MDA MB 231 cells. For HEK293T, ***$P = 6.05*10^{-5}$, MCF10a, **$P = 0.008$, and MDA MB 231, *$P = 0.012$, unpaired t test. Data are mean±SD of biological replicates, n = 3/group. (**D**) TNBC risk association with the risk allele of rs57141087 using GWAS data for African ancestry (Jia et al, 2024). (**E**) Luciferase reporter assays measuring the enhancer activity of DNA fragments (Chr17: 59,107,961–59,107,053) in HEK293T cells. The data is normalized to the vector only set to 1 and presented as a fold change in luminescence. of the different fragments in HEK293T. For 353 bp, ***$P = 0.0001$, 909 bp, *$P = 0.012$, and 353 bp vs. 909 bp, $P = 0.539$, unpaired t test. Data are mean±SD of biological replicates, $n = 3$/group.

© The Author(s)

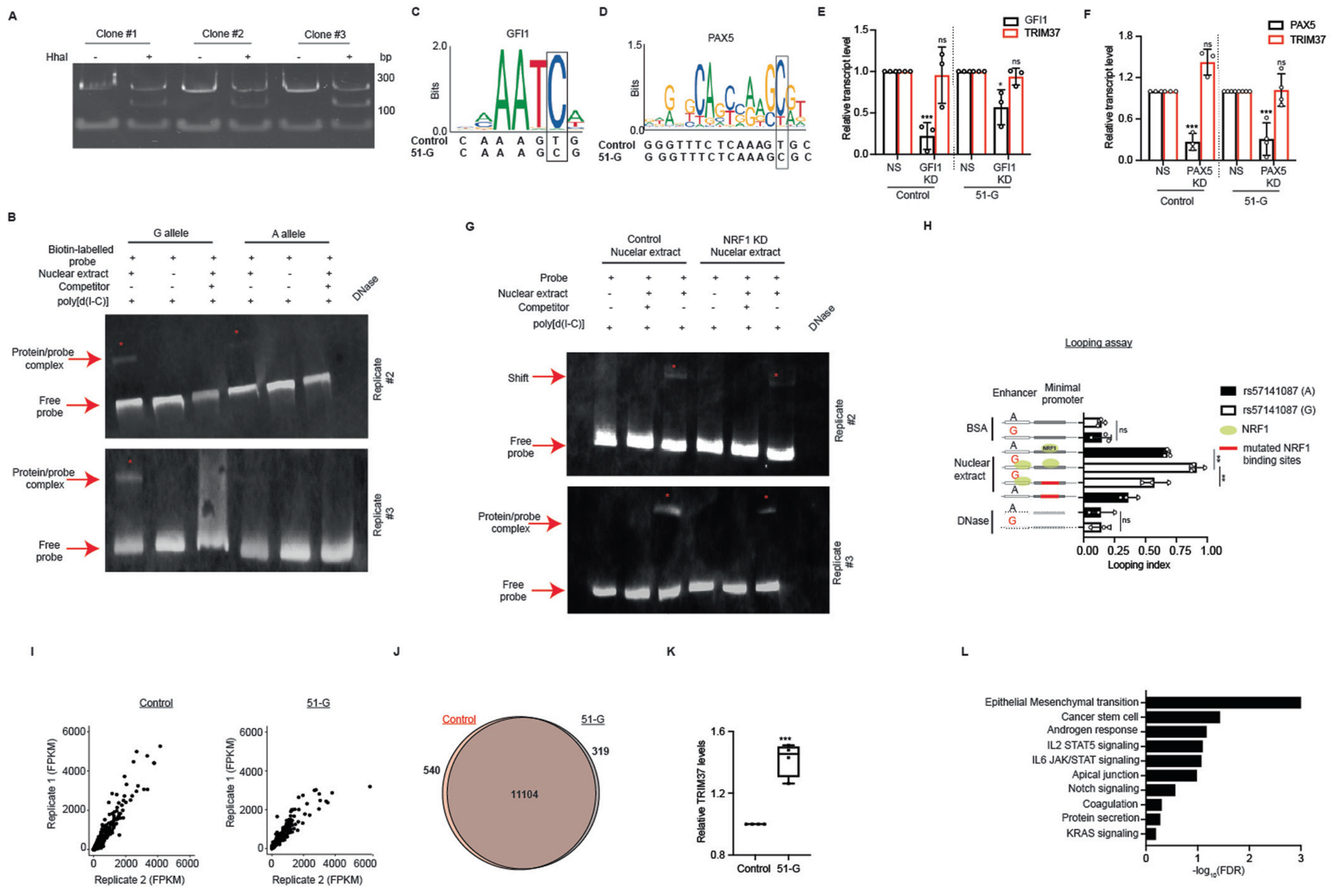

Figure EV4. NRF1 binds uniquely to the enhancer region harboring the rs57141087 risk allele.

(**A**) Representative polyacrylamide gel indicates a distinct HhaI digestion pattern for KTB51 clones harboring rs57141087 reference (A) and risk (G) alleles. The data for three different clones is shown. (**B**) Immunoblot for biotin-labeled TRIM37 enhancer fragment with risk (G allele) and reference (A allele) incubated with HEK293T nuclear extract and competitor (unlabeled probe) as indicated. The bound and free DNA fragments are indicated with red arrows, and the shift in the band is indicated (red asterisk, *). The concentrations of nuclear extract (5 µg), Biotin-labeled probes (0.2 pmol), competitor (10 pmol), and poly [d(I-C)] (50 ng/µl) were indicated at the *Top*. The representative images from two different experiments (Replicate #1–2) are shown. (**C**, **D**) The risk G allele of rs57141087 harboring GFI1 (**C**) and PAX5 (**D**) binding motif in the enhancer region of TRIM37 (Chr17: 59,107,267–59,107,262) is shown. (**E**, **F**) qRT-PCR monitoring *TRIM37* levels in GFI1 (**E**) and PAX5 (**F**) knockdown in KTB51 cells with A or G allele for rs57141087. Gapdh is used as an endogenous control. For GFI1 knockdown, GFI1 (A), $^{***}P = 0.001$, TRIM37 (A), $^{ns}P = 0.831$, GFI1 (**G**), $^{*}P = 0.024$, and TRIM37 (G), $^{ns}P = 0.345$. For PAX5 knockdown, PAX5 (A), $^{***}P = 0.0005$, TRIM37 (A), $^{ns}P = 0.187$, PAX5 (**G**), $^{***}P = 0.001$, and TRIM37 (**G**), $^{ns}P = 0.874$, unpaired *t* test. Data are mean±SD of biological replicates, $n \geq 3$/group. (**G**) Immunoblot for biotin-labeled TRIM37 enhancer fragment incubated with nuclear extract from control or NRF1 knockdown HEK293T cells and unlabeled probe (competitor) as indicated. The bound and free DNA fragments are indicated with red arrows, and the shift in the band is indicated (red asterisk, *). The concentrations of nuclear extract (5 µg), Biotin-labeled probes (0.2 pmol), competitor (10 pmol), and poly [d(I-C)] (50 ng/µl) were indicated at the *Top*. The representative images from two different experiments (Replicate #1–2) are shown. (**H**) A looping assay measuring the promoter–enhancer interactions for in vitro synthesized DNA fragments (Chr17: 59,107,405-59,106,446) with risk or reference alleles or mutated NRF1 binding motifs in the promoter was used. For BSA, $^{ns}P = 0.947$, NRF1, $^{**}P = 0.003$, NRF1 vs mutated NRF1, $^{**}P = 0.009$, and DNase, $^{ns}P = 0.965$, unpaired *t* test. Data are mean±SD of biological replicates, $n = 3$/group. (**I**) Correlation plots of the FPKM read from RNA-seq analysis for each replicate are shown for control (*Left*) and 51-G (*Right*) cells. (**J**) Venn diagram showing the overlap between differentially expressed genes in control and 51-G cells. (**K**) qRT-PCR analysis monitoring *TRIM37* in control and 51-G cells. Gapdh is used as an endogenous control. $^{***}P = 0.0003$, unpaired t test. Data are mean ± SD of biological replicates, $n = 4$/group. The boxed areas span the first to the third quartile. The whiskers represent the 15th and 85th percentiles. (**L**) The top ten pathways enriched in 51-G relative to control cells identified by GSEA are shown.

 © The Author(s)

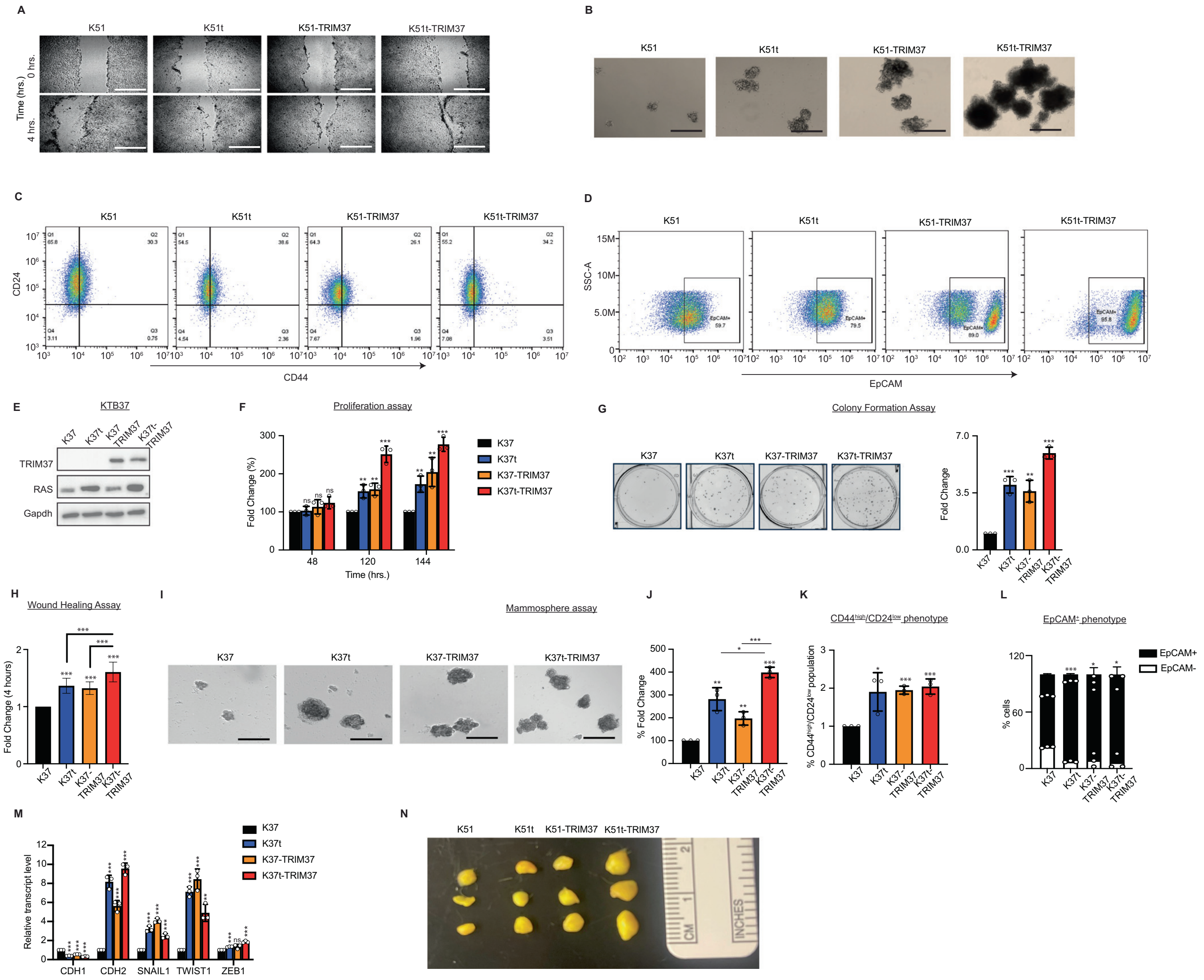

Figure EV5. ***TRIM37*-directs neoplastic transformations in immortalized epithelial cells.**

(**A**) Representative phase contrast images of relative migratory abilities for KTB51, K51t, K51-TRIM37, and K51t-TRIM37 after 4 h. (10X, scale bar, 300 µm). (**B**) Representative images of solid mammospheres formed by KTB51, K51t, K51-TRIM37, and K51t-TRIM37 cells. (10X, scale bar, 300 µm). (**C, D**) Representative FACS plot showing gating strategy and distribution of stained population for CD24 and CD44 (**C**) and EpCAM (**D**) in KTB51, K51t, K51-TRIM37, and K51t-TRIM37 cells. (**E**) Immunoblots in KTB37 and *TRIM37* derivatives of RAS-transformed KTB37(K37t) cells. Gapdh was the loading control. (**F**) Relative cell growth for KTB37 and *TRIM37* derivatives of RAS-transformed KTB37 (K37t-TRIM37) cells at indicated times. For 48 h., K37t, $^{ns}P = 0.655$, K37-TRIM37, $^{ns}P = 0.287$, and K37t-TRIM37, $^{ns}P = 0.058$. For 120 h., K37t, $^{**}P = 0.006$, K37-TRIM37, $^{**}P = 0.003$, and K37t-TRIM37, $^{***}P = 0.0003$. For 144 h. K37t, $^{**}P = 0.004$, K37-TRIM37, $^{**}P = 0.009$, and K37t-TRIM37, $^{***}P = 0.0002$, unpaired *t* test. Data are mean ± SD of biological replicates, $n = 3$/group. (**G**) Representative bright-field images after crystal violet staining show the growth of KTB37, K37t, K37-TRIM37, and K37t-TRIM37 cells. The colonies were quantified (*Right*). For K37t, $^{***}P = 0.0005$, K37-TRIM37, $^{**}P = 0.003$, and K37t-TRIM37, $^{***}P = 1.86{*}10^{-5}$, unpaired t test. Data are mean ± SD of biological replicates, $n = 3$/group. (**H**) The relative migratory abilities for KTB37, K37t, K37-TRIM37, and K37t-TRIM37 cells were quantitated after 4 h. For K37t, $^{***}P = 1.32{*}10^{-13}$, K37-TRIM37, $^{***}P = 3.39{*}10^{-14}$, and K37t-TRIM37, $^{***}P = 1.72{*}10^{-16}$; K37t vs. K37t-TRIM37, $^{***}P = 4.13{*}10^{-5}$, K37-TRIM37 vs. K37t-TRIM37, $^{***}P = 1.28{*}10^{-6}$, unpaired t test. Data are mean±SD of biological replicates, $n = 6$/group. (**I, J**) Representative images (10X, scale bar, 300 µm) (**I**) and quantitation (**J**) of solid mammospheres formed by KTB37, K37t, K37-TRIM37, and K37t-TRIM37 cells. The colonies were quantified. For K37t, $^{**}P = 0.003$, K37-TRIM37, $^{**}P = 0.004$, and K37t-TRIM37, $^{***}P = 2.15{*}10^{-5}$; K37t vs. K37t-TRIM37, $^{*}P = 0.021$, K37-TRIM37 vs. K37t-TRIM37, $^{***}P = 0.0007$, unpaired t test. Data are mean±SD of biological replicates, $n = 3$/group. (**K, L**) FACS analysis of CD24 and CD44 (**K**) and EpCAM (**L**) in KTB37, K37t, K37-TRIM37, and K37t-TRIM37 cells derived from mammospheres in (**I, J**). For $CD44^{high}/CD24^{low}$ in K37t, $^{*}P = 0.036$, K37-TRIM37, $^{***}P = 0.0001$, and K37t-TRIM37, $^{***}P = 0.0008$. For $EpCAM^{+}$ in K37t, $^{***}P = 1.99{*}10^{-5}$, K37-TRIM37, $^{*}P = 0.028$, and K37t-TRIM37, $^{*}P = 0.024$, unpaired t test. Data are mean±SD of biological replicates, $n = 3$/group. (**M**) qRT-PCR analysis of EMT markers in KTB37, K37t, K37-TRIM37, and K37t-TRIM37 cells derived from mammospheres in (**I, J**). Gapdh is used as an endogenous control. For CDH1 in K37t, $^{***}P = 2.03{*}10^{-7}$, K37-TRIM37, $^{***}P = 1.01{*}10^{-6}$, and K37t-TRIM37, $^{***}P = 1.58{*}10^{-5}$. For CDH2 in K37t, $^{***}P = 5.52{*}10^{-5}$, K37-TRIM37, $^{***}P = 0.0001$, and K37t-TRIM37, $^{***}P = 1.38{*}10^{-5}$. For SNAI1 in K37t, $^{***}P = 0.0002$, K37-TRIM37, $^{***}P = 2.56{*}10^{-5}$, and K37t-TRIM37, $^{***}P = 0.0006$. For TWIST1 in K37t, $^{***}P = 3.08{*}10^{-5}$, K37-TRIM37, $^{***}P = 0.0003$, and K37t-TRIM37, $^{**}P = 0.002$. For ZEB1 in K37t, $^{***}P = 0.0001$, K37-TRIM37, $^{ns}P = 0.051$, and K37t-TRIM37, $^{***}P = 0.0002$, unpaired t test. Data are mean±SD of biological replicates, $n = 3$/group. (**N**) Representative bright-field images showing gross histology of xenografts harvested from NSG mice injected with, K51t, K51-TRIM37, and K51t-TRIM37 cells.

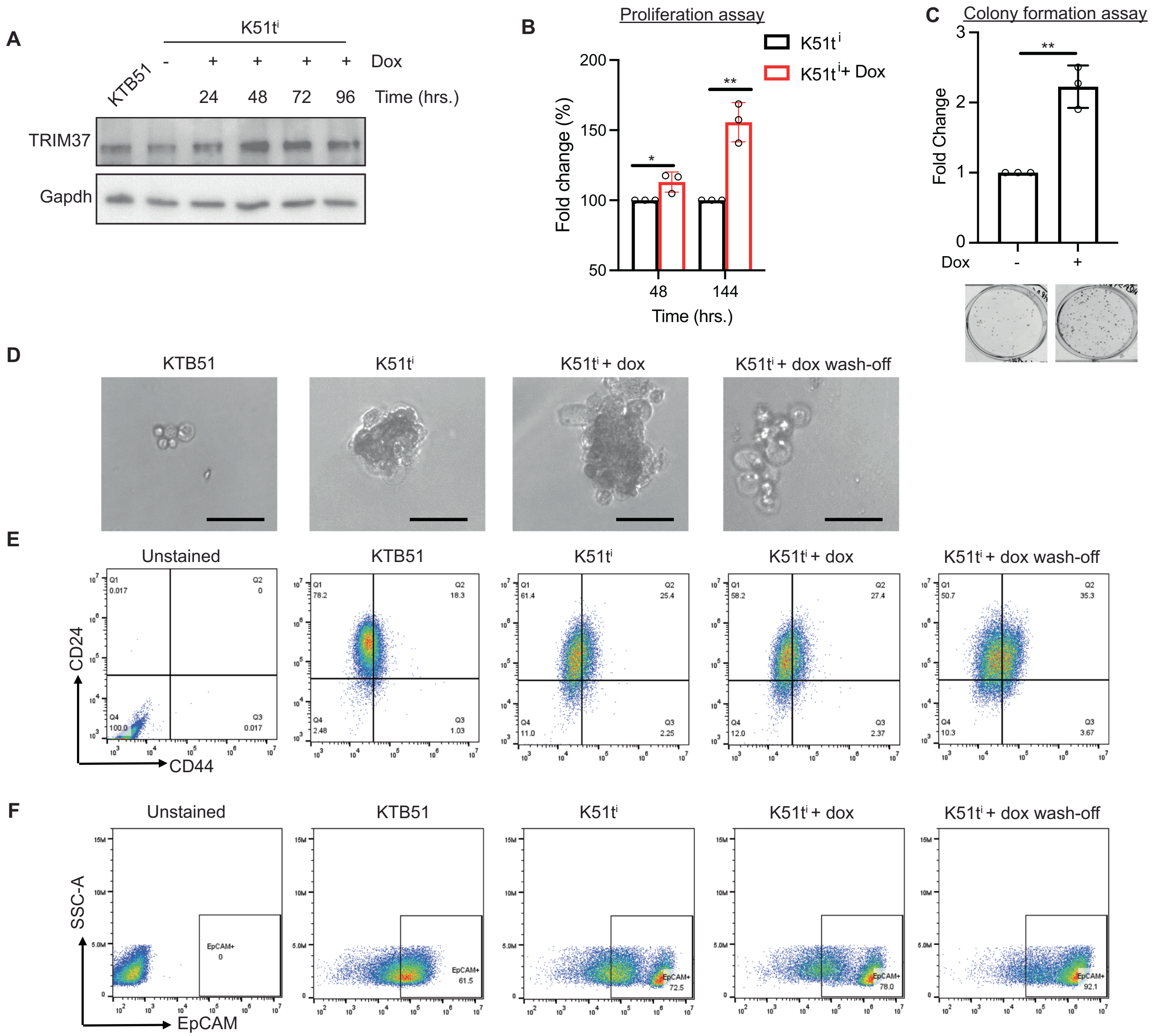

Figure EV6. TRIM37 is required for breast cancer onset.

(**A**) Immunoblot analysis in K51t^i cells following doxycycline (dox) treatment for 24, 48, 72 and 96 h. post-induction. Gapdh was the loading control. (**B**) Relative cell growth for K51t^i and dox-induced K51t^i at indicated times. For 48 h, $^*P = 0.034$ and 144 h, $^{**}P = 0.002$, unpaired t test. Data are mean±SD of biological replicates, $n = 3$/group. (**C**) The colony formation assay quantitating the growth of K51t^i and dox-induced K51t^i. Representative bright-field images after crystal violet staining are shown (*Bottom*). For K51t^i vs. K51t^i+dox, $^{**}P = 0.003$, unpaired t test. Data are mean±SD of biological replicates, $n = 3$/group. (**D**) Representative images of solid mammospheres formed by K51t^i and dox-induced K51t^i at indicated times. (×10, scale bar, 300 μm). (**E, F**) Representative FACS plot showing gating strategy and distribution of stained population for CD24 and CD44 (**E**) and EpCAM (**F**) in K51t^i and dox-induced K51t^i at indicated times.

© The Author(s)
